# Delayed Identification of Cortical Superficial Siderosis in a Patient with Recurrent Transient Focal Neurological Symptoms: A Case Report

**DOI:** 10.7759/cureus.5211

**Published:** 2019-07-23

**Authors:** Kristina A Rankine, Asia Filatov, Pamraj Sharma, Kettia Alusma- Hibbert, Patricio S Espinosa

**Affiliations:** 1 Neurology, Charles E. Schmidt College of Medicine, Florida Atlantic University, Boca Raton, USA; 2 Neurology, Marcus Neuroscience Institute - Boca Raton Regional Hospital, Boca Raton, USA

**Keywords:** superficial siderosis, amyloid angiopathy, intracerebral hemorrhage, subarachnoid hemorrhage, boston criteria

## Abstract

Cortical superficial siderosis (cSS), also referred to as sulcal siderosis, is a neurological condition characterized by hemosiderin subpial deposits in the cortical sulci over the convexities of cerebral hemispheres. These deposits are further found sparingly in the spinal cord, brainstem, and cerebellum. Patients typically present with transient focal neurological symptoms that make cSS challenging to differentiate from other acute neurological processes such as transient ischemic attacks (TIA), focal seizures, and acute convexity subarachnoid hemorrhage (cSAH). This condition is presently recognized as a characteristic feature of the age-associated disorder referred to as cerebral amyloid angiopathy (CAA). This paper describes a patient who presented with transient neurologic symptoms, first suspected to be secondary to acute subarachnoid hemorrhage (SAH), found to have cSS and cerebral amyloid angiopathy.

## Introduction

Siderosis, which is obtained from *sideros*, a Greek word, refers to the accumulation of compounds containing iron in body tissues. Superficial siderosis occurs when blood breakdown products such as iron and hemosiderin are deposited in the pial and subpial regions of the brain. Superficial siderosis is further subclassified into classical superficial siderosis with clinical features including ataxia, sensorineural hearing loss and cognitive decline that is attributed to blood residue deposition primarily in the infratentorial regions and spinal cord [1-2]. A second form called cortical superficial siderosis (cSS) has been recently described as siderosis limited to the supratentorial region and the convexities of cerebral hemispheres, that may manifest as episodes of transient focal neurological episodes. It may be an indicator of increased future intracerebral hemorrhage (ICH) risk in patients with cerebral amyloid angiopathy (CAA) [2]. Notably, the most common clinical manifestation of CAA is spontaneous ICH. Despite this possible risk association, cSS itself is a separate entity that may resemble subarachnoid hemorrhage on brain imaging. Further, cSS has significant relevance in neurovascular, epilepsy, and memory-based services.

Clinical studies have demonstrated that cSS is caused by repeated episodes of convexity subarachnoid hemorrhage (cSAH) from weakened leptomeningeal vessels affected by CAA. As acute blood products are gradually degraded, there is a deposition of residues, such as hemosiderin, in the superficial cortical layers. The neuropathologic mechanisms underlying cSS remain unclear [3-4]. The ability to identify cSS may prove helpful in risk stratification of patients and further clinical practice and management, especially regarding antithrombotic and anticoagulant use. The most common diagnosis and assessment of superficial siderosis is magnetic resonance imaging (MRI) where the folia and cerebellar vermis are the best areas for locating the deposition. In this paper, we describe the case of an individual who presented with transient neurologic symptoms initially suspected to be secondary to acute SAH found to have cSS and CAA.

## Case presentation

A 75-year-old right-handed female with no significant past medical history presented to an outside facility after having several episodes of right arm numbness and weakness. Additionally, perioral numbness and occasional word-finding difficulties prompted an MRI of the brain, which revealed a left frontal convexity and right parietal convexity SAH. The patient underwent catheter cerebral angiography that showed no evidence of aneurysm. She was initiated on Keppra for presumed focal seizures.

**Figure 1:**
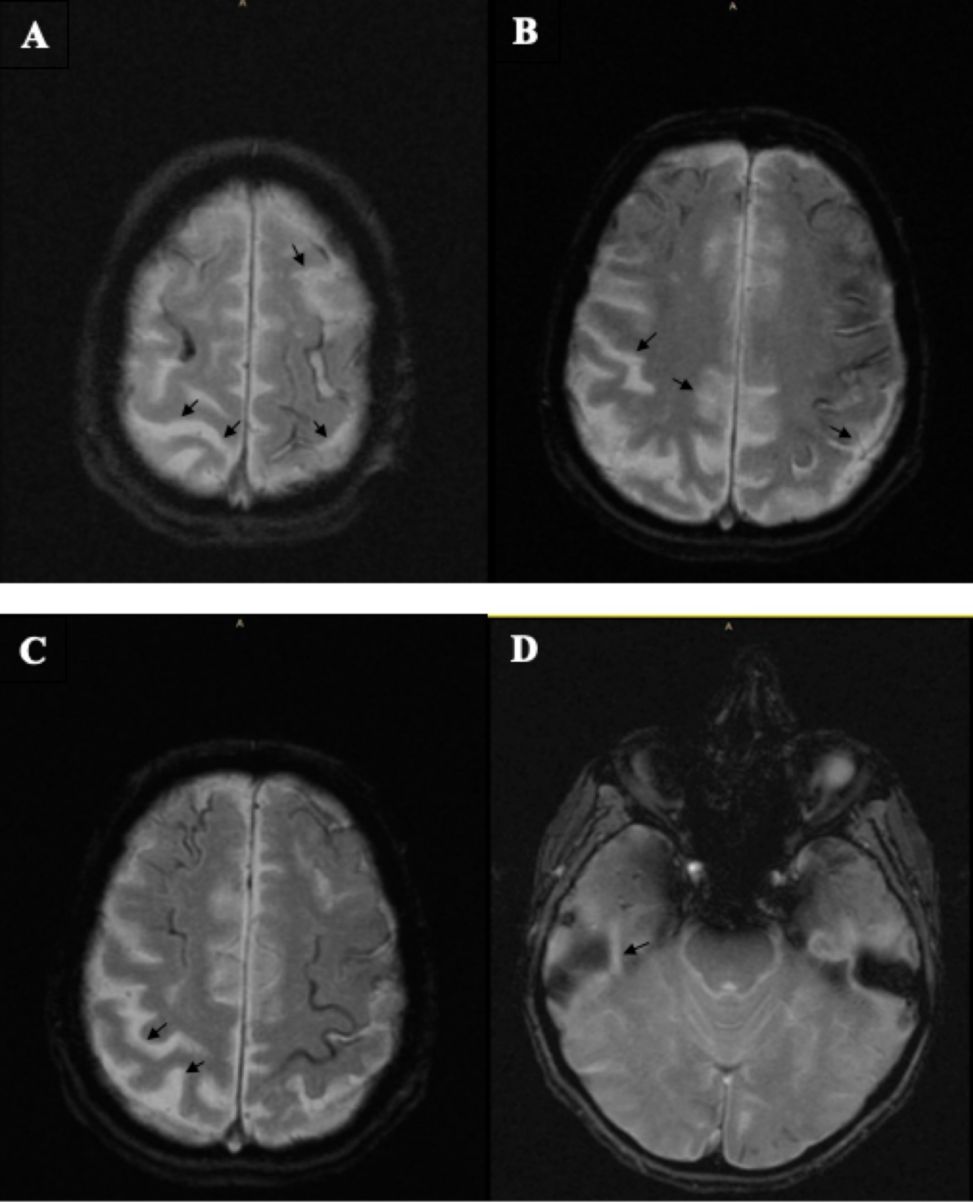
Detection of cortical superficial siderosis on MRI Distribution of cSS is supratentorial with no intraparenchymal involvement as is seen in ICH. A. cSS; B. bilateral temporal; C. left temporal; D. right temporal and left occipital lobes cSS, cortical superficial siderosis; ICH, intracerebral hemorrhage

After discharge, the patient had recurrent symptoms, and another brain MRI showed no new areas of SAH. Neurosurgery was consulted for suspected aneurysms, and a cerebral angiogram was carried out, which showed no acute pathology. MRI of the brain revealed bilateral cortical superficial siderosis with associated bilateral occipital, posterior Sylvian and left temporal siderosis (Figure 1 A-D). There were also microbleeds from areas of amyloid angiopathy in the left internal capsule, which were consistent with her right-sided symptoms, and it was determined that the patient’s transient recurrent neurological clinical presentations were the sequelae of her underlying amyloid angiopathy and cortical superficial siderosis. The patient was advised to refrain from anticoagulant and antiplatelet use due to an increased risk of intracerebral hemorrhaging in the setting of both amyloid angiopathy and aneurysm.

## Discussion

The clinical relevance of cSS is still being investigated, but its association with CAA and ICH risk is becoming better understood. A recent study demonstrated that cSS was found by MRI investigation in 44.1% of patients with probable CAA based on the modified Boston criteria (Table 1) [5], that presented with cognitive impairment and transient focal neurological symptoms. Further, cSS progression was found to independently predict increased symptomatic ICH risk [6].

**Table 1:** Modified Boston Criteria CAA, cerebral amyloid angiopathy

	Modified Boston Criteria
Definite CAA	Full post-mortem examination demonstrating:
	- Lobar, cortical or corticosubcortical hemorrhage
	- Severe CAA with vasculopathy
	- Absence of another diagnostic lesion
Probable CAA with supporting pathology	Clinical data and pathological tissue (evaluated) hematoma or cortical biopsy) demonstrating:
	- Lobar, cortical or corticosubcortical hemorrhage
	- Some degree of CAA in the specimen
	- Absence of another diagnostic lesion
Probable CAA	Clinical data and MRI or CT demonstrating:
	- Multiple hemorrhages restricted to lobar, cortical or corticosubcortical regions
	- Single lobar, cortical, or corticosubcortical hemorrhage and focal or disseminated superficial siderosis
	- Age ≥55
	- Absence of other cause of hemorrhage or superficial siderosis
Possible CAA	Clinical data and MRI or CT demonstrating:
	- Single lobar, cortical or corticosubcortical hemorrhage, or
	- Focal or disseminated superficial siderosis
	- Age ≥55
	- Absence of other cause of hemorrhage or superficial siderosis

As cSS is most often found in patients that present with transient focal neurological episodes, that may be attributed to TIA, the ability to differentiate these entities is paramount [6-7]. Patients thought to have TIA are often placed on antithrombotics for secondary stroke prevention and given the aforementioned increased ICH risk in patients with cSS, this management approach would be suboptimal as it would increase the risk of bleeds and potential patient morbidity.

Though cSS association with CAA and ICH risk is being studied, there have not been numerous investigations into cSS being misidentified as acute cSAH or how this impacts patient management or outcomes. As is exemplified with our patient, cSS being attributed to an acute cSAH may prompt unnecessary interventions for patients. For example, cerebral angiography is often undertaken to find a source of bleeding, but this is typically unrewarding possibly due to the slow and intermittent bleeding associated with superficial siderosis in general or the chronic ongoing resolution of a prior bleed [8]. Drawing from the pathophysiology of cSS, a single hemorrhage is insufficient to cause the condition, thus profound diagnosis should be conducted including establishing the location of the deposition. Therefore, it is essential to be able to identify cSS in patients when evaluating their ischemic stroke or hemorrhage risk and developing management plans. Effective diagnosis and management will enhance the quality of patient outcomes and increase patient safety. 

The clinical presentations of patients with cSS are typically recurrent transient focal neurological episodes, as was the case with our patient. These presentations are characterized as lasting from minutes to hours and include episodes of paresthesias, numbness, or weakness that typically resolve over a short time period. Essential to note is that these symptoms are distinct from those of the typical SAH patient who presents with the characteristic thunderclap headache. Currently, it is difficult to distinguish the frequency of patients presenting with transient focal neurological episodes that are secondary to CAA, cSS, or cSAH versus a transient ischemic attack (TIA) as few centers routinely obtain blood-sensitive MRI sequences which are necessary to detect these underlying processes [6]. Further, since cSS is not a commonly diagnosed radiographic finding, a better understanding of distinguishing characteristics, as shown in Table 2, could increase the likelihood of cSS being identified. The MRI appearance of cSS is a result of deposition of blood breakdown products that result in a curvilinear “track-like” pattern along the cortical surface [9].

**Table 2:** Differences in presentation of cSS and acute cSAH on MRI *cSS is not typically detected on T1 weighted imaging unless there has been a subacute bleed [6]. **Blood breakdown residues cause local magnetic field inhomogeneity that leads to loss of signal in T2 and SWI sequences [7]. cSS, cortical superficial siderosis; cSAH, convexity subarachnoid hemorrhage; SWI, susceptibility-weighted imaging

MRI Mode	cSS	Acute cSAH
T1	Low signal*	High signal
T2	Low signal**	High signal
Gradient Echo	Low signal with blooming	High signal
Susceptibility Weighted Imaging	Low signal with blooming**	High signal
Location of blood products	- Superficial layers of the cerebral cortex - Subarachnoid Space	Subarachnoid Space

In patients such as ours, with recurrent unexplained transient focal neurological episodes, it could be beneficial in evaluation to obtain blood-sensitive MRI sequences to elucidate the etiology of the patients’ symptoms. Additionally, future studies into the pathophysiology, pathogenesis, and progression of cSS, as well as accumulation of evidence-based data to guide therapy for patients found to have cSS will influence our ability to risk-stratify patients as well as inform our use of antithrombotic and anticoagulant agents in developing effective and patient-centered interventions for the management of these patients.

## Conclusions

cSS is characterized by blood breakdown products, such as hemosiderin, being deposited in the cortical sulci. It is difficult to identify because it presents like other neurological processes with transient focal neurological symptoms and requires blood-sensitive MRI for an accurate diagnosis. Given recent studies demonstrating the association of cSS with intracranial hemorrhage, a better understanding of the mechanisms of cSS will influence our treatment approach, especially in the utilization of antithrombotic and anticoagulant agents for improved patient outcomes.
